# Qualitative differences in brain-infiltrating T cells are associated with a fatal outcome in mice infected with Japanese encephalitis virus

**DOI:** 10.1007/s00705-014-2154-8

**Published:** 2015-01-22

**Authors:** Kenji Shirai, Daisuke Hayasaka, Kazutaka Kitaura, Tomohiko Takasaki, Kouichi Morita, Ryuji Suzuki, Ichiro Kurane

**Affiliations:** 1 Department of Virology 1, National Institute of Infectious Diseases, Tokyo, 162-8640 Japan; 2 Department of Rheumatology and Clinical Immunology, Clinical Research Center for Allergy and Rheumatology, Sagamihara National Hospital, National Hospital Organization, Kanagawa, 252-0392 Japan; 3 Department of Infection Biology, Institute of Basic Medical Sciences, University of Tsukuba, Ibaraki, 305-8575 Japan; 4 Department of Virology, Institute of Tropical Medicine, GCOE program, Nagasaki University, Nagasaki, 852-8523 Japan

## Abstract

Japanese encephalitis (JE) is the most important form of viral encephalitis in Asia. The critical factors determining mortality and severity of JE virus (JEV) infection remain unclear. We identified brain-infiltrating T cells associated with a fatal outcome of JEV infection in mice. Dying mice were defined as those that lost more than 25 % of their body weight by day 13 and died by day 21, while surviving mice were defined as those that lost less than 10 % by day 13, based on the result of the survival time course study. Two groups of five mice that demonstrated brain virus titers of >1 × 10^6^ pfu/g were randomly selected from the dying and surviving groups and used in the analyses. Cytokine patterns in brains were first examined, revealing a higher ratio of Th1-related cytokine genes in dying mice. The expression levels of CD3, CD8, CD25, and CD69 increased in JEV-infected mice relative to mock-infected mice. However, expression levels of these cell-surface markers did not differ between the two groups. T-cell receptor (TCR) usage and complementary determining region 3 (CDR3) sequences were analyzed in the brain-infiltrating T cells. T cells expressing VA8-1, VA10-1, and VB2-1 increased in both groups. However, the dominant T-cell clones as defined by CDR3 amino acid sequence differed between the two groups. The results indicate that the outcome of JEV infection, death or survival, was determined by qualitative differences in infiltrating T-cell clones with unique CDR3 amino acid sequences.

## Introduction

Japanese encephalitis virus (JEV) is a member of the family *Flaviviridae.* JEV is endemic in many countries located in Southeast and South Asia [1]. JEV causes fatal encephalitis associated with damage to the central nervous system (CNS) in humans. Clinical manifestations caused by JEV range from infections and fevers, with complete patient recovery, to debilitating or fatal encephalitis. The fatality rate is as high as 20–30 %, and neurological sequelae are observed in about 50 % of surviving patients [2]. JEV strain JaOArS982 has an approximately 30 % mortality rate in mice over a wide dose range (10^4^–10^6^ PFU) following subcutaneous inoculation [3]. Although a dose-independent mortality pattern has been reported in mouse models of encephalitic flavivirus infections, the viral and immunological mechanisms that determine fatality or survival have yet to be defined [4–8].

Multiple factors are associated with encephalitis pathogenesis. It is believed that neutralizing antibodies play a critical role in protection from JEV, and brain-infiltrating T cells play an important role in the pathogenesis and recovery from viral encephalitis [5, 6, 8, 9]. Experiments using knockout mice or passive cell transfer at the polyclonal level suggest that cytotoxic T lymphocytes (CTL) play a role in the protection and recovery from JEV and other flavivirus infections [10–16]. T cells potentially contribute to both recovery and immunopathogenesis, and the functional balance is affected by viral species and/or experimental conditions. For example, reports indicate that T-cell responses are essential for viral clearance in WNV infection [15, 17–20], although differences in responses between surviving and dying mice under identical inoculation conditions have not been determined. Using T cell receptor (TCR) repertoire analysis and nucleotide sequencing of the complementary-determining region 3 (CDR3), we previously demonstrated that selected TCRs accumulate in JEV-infected mouse brain [21]. We therefore attempted to define the pathological and/or protective mechanism in our JEV-infected mouse model by analyzing the relative expression levels of each TCR family and the T-cell clone frequency.

In the present study, we compared the TCR repertoire and T-cell clone frequency between surviving and dying mice. Identical patterns would suggest that disease severity is independent of T cells, whereas different patterns would suggest that T-cell antigen recognition patterns are related to the infection outcome. We sought to determine whether infection outcomes, death and survival, are determined by these qualitative or quantitative differences in infiltrating T cells.

## Materials and methods

### Ethics statement

The animal experiments were performed in accordance with the recommendations in the ARRIVE guidelines (http://www.nc3rs.org.uk/page.asp?id=1357) and Fundamental Guidelines for Proper Conduct of Animal Experiment and Related Activities in Academic Research Institutions under the jurisdiction of the Ministry of Education, Culture, Sports, Science and Technology (http://www.mext.go.jp/b_menu/hakusho/nc/06060904.htm). The experimental protocols were approved by the Animal Care and Use Committee of Nagasaki University (approval number: 091130-2-7 / 0912080807-7).

### Virus

The JEV JaOArS982 strain (GenBank accession no. M18370) exhibits mild pathogenicity relative to JaTH160 (GenBank accession no. AB269326) in the mice used in this study [22]. The virus was obtained from the cell culture medium of baby hamster kidney (BHK) cells infected with the virus previously prepared in suckling mouse brains. The BHK cells were maintained in Eagle’s minimal essential medium (EMEM; Nissui Pharmaceutical Co.) containing 8 % fetal calf serum (FCS) and antibiotics.

### Infection of mice with JEV

C57BL/6j (B6) female mice (Japan SLC, Inc., Hamamatsu, Japan) were kept in a specific-pathogen-free environment. Seven-week-old B6 mice were injected subcutaneously (s.c.) with 10^4^ PFU/0.5 ml of JEV diluted in EMEM containing 2 % FCS. Mock-infected mice were inoculated with EMEM from supernatants of BHK cells. Day 0 was defined as the day of JEV inoculation. Mock- or JEV-infected mice were euthanized under anesthesia with isoflurane on day 13.

### Selection of mice

After inoculation of mice with JEV, body weights were examined daily from day 1 to day 21. The levels of the change in the body weights differed among inoculated mice. Approximately one-fourth of JEV-infected mice demonstrated weight loss of more than 25 % by day 13. These mice started to die on day 14, and all died by day 21. None of the mice that lost less than 10 % of their body weight by day 13 died during the observation period. The group of mice that lost between 10 and 25 % of their body weight by day 13 included some that died by day 21 and some that survived. These results indicated that body weight loss of more than 25 % at day 13 is an indicator of a fatal outcome under these experimental conditions. Because some of the mice started to die on day 14, day 13 was the last day on which all of the mice inoculated with JEV were still alive, allowing the outcome (survival or death) of an individual mouse to be predicted based on body weight. We therefore selected day 13 as the time point to determine whether the outcome would be fatal and to analyze T cell responses.

Based on these results, two groups of mice, the dying mouse group and the surviving mouse group, were defined and used in the experiments. Dying mice were defined as those that lost more than 25 % of their body weight by day 13, and surviving mice were defined as those that lost less than 10 % of their body weight by day 13. The mice that lost 10–25 % of their body weight by day 13 were not used in the experiments because it was not possible to predict the outcome of these mice between days 14 and 21, with some mice dying and some surviving. JEV levels in brains were measured in plaque-forming units on day 13. The levels were higher than 1 × 10^6^ pfu/g in all mice categorized as dying mice. The levels were variable in those categorized as surviving mice; some demonstrated JEV levels higher than 1 × 10^6^ pfu/g, but the others demonstrated low levels. Five mice were selected randomly from the dying mouse group, and all of these mice had a JEV titer of >1 × 10^6^ pfu/g. Five mice were also randomly selected from those that were defined as surviving mice and had a JEV titer of >1 × 10^6^ pfu/g. We thus were able to compare two groups of five mice each of which had a brain JEV titer of >1 × 10^6^ pfu/g, regardless of whether they belonged to the dying or the surviving group.

### Isolation of total RNA

Mock- or JEV-infected mice were euthanized and perfused with cold PBS at 13 dpi. Brains and spleens were excised and immediately submerged in RNA*later* ^®^ RNA stabilization reagent (QIAGEN, Hilden, Germany) [23]. Total RNA was isolated using an RNeasy Lipid Tissue Mini Kit (QIAGEN) according to the manufacturer’s instructions. Isolated total RNA was used for quantification of viral RNA and gene expression using quantitative real-time PCR, TCR repertoire analysis, and CDR3 sequencing.

### Quantitative real-time PCR (qRT-PCR)

Expression levels of T-cell-related antigens (CD3, CD4, CD8, CD25, and CD69), cytokines (IL-4, IL-5, TNF-α, and IFN-γ), apoptosis-related genes (granzyme (Gzm) A, Gzm B, perforin, Fas ligand (FasL)), and regulatory T (Treg) cell related factor (transforming growth factor beta 1 (TGF-β1) were determined using qRT-PCR. The primers used were as follows: TGF-β1 (forward, 5′-GTGTGGAGCAACATGTGGAACTCTA-3′; reverse, 5′-CGCTGAATCGAAAGCCCTGTA-3′), forkhead/winged helix transcription factor 3 (Foxp3) (forward, 5′-CTCATGATAGTGCCTGTGTCCTCAA-3′; reverse, 5′-AGGGCCAGCATAGGTGCAAG-3′), transcription factor (T-bet (forward, 5′-AGGCTGCCTGCAGTGCTTCTA-3′; reverse, 5′-GGACACTCGTATCAACAGATGCGTA-3′), and GATA-3 (forward, 5′-ATGGTACCGGGCACTACCTTTG-3′; reverse, 5′-TGACAGTTCGCGCAGGATG-3′). qRT-PCR was performed using a Bio-Rad CFX96 system (Bio-Rad Laboratories, Inc., Hercules, CA, USA) for brains excised from mock- or JEV-infected mice. The sequences of the specific primer pairs were reported previously [21, 24]. The housekeeping gene encoding glyceraldehyde 3-phosphate dehydrogenase (GAPDH) was used as an internal control. Freshly isolated RNA was converted to cDNA using a PrimeScript™ RT Reagent Kit (Takara Bio Inc., Shiga, Japan), qRT-PCR was performed using SsoFast™ EvaGreen^®^ Supermix (Bio-Rad) according to the manufacturer’s instructions, and expression levels were measured as reported previously [25]. The absolute copy number was calculated using a standard curve generated by serial dilution (10^1^–10^8^ copies) of a recombinant plasmid encoding each gene of interest.

Viral RNA levels of JEV were examined using specific primers for the JEV envelope protein gene (forward, 5′-ATGACCTCGCTCTCCCCTGG-3′; reverse, 5′-GACCCAAGAGCAACAACGGA-3′). Reverse transcription and qRT-PCR reactions were conducted as described above. Viral RNA was quantified as the copy number per 1 ng of total RNA. Except for viral RNA, copy numbers were normalized based on the copy number of the housekeeping gene GAPDH.

### Adaptor-ligation-mediated polymerase chain reaction (AL-PCR)

AL-PCR methodology has been reported previously [26–28]. Briefly, isolated total RNA was converted to double-stranded cDNA using a Superscript cDNA Synthesis Kit (Invitrogen, Carlsbad, CA, USA) according to the manufacturer’s instructions, except that a specific primer (BSL-18E) was used [28]. The P10EA/P20EA adaptors were ligated to the 5′ end of cDNA, and the adaptor-ligated cDNA was digested with *Sph* I. PCR was performed using TCR α-chain or β-chain constant-region-specific primers (MCA1 or MCB1) and P20EA. The second PCR was performed with MCA2 or MCB2 and P20EA. Biotinylation of PCR products was performed using P20EA and 5′-biotinylated MCA3 or MCB3 primers. Consistencies in the results between the PCR assay and protein detection assay have been confirmed and reported [28].

### TCR repertoire analysis

Ten picomoles of amino-modified oligonucleotides specific for the TCR α-chain variable (TCRAV) and TCR β-chain variable (TCRBV) segments were immobilized onto carboxylate-modified 96-well microplates with water-soluble carbodiimide. Prehybridization and hybridization were performed in GMCF buffer (0.5 M Na_2_HPO_4_, pH 7.0, 1 mM EDTA, 7 % SDS, 1 % BSA, and 7.5 % formamide) at 47 °C. One hundred microliters of the denatured 5′-biotinylated PCR product was mixed with an equal volume of 0.4 M NaOH/10 mM EDTA, and the mixture was added to 10 ml GMCF buffer. One hundred microliters of hybridization solution was used in each microplate well containing immobilized V-segment-specific oligonucleotide probes. After hybridization, wells were washed four times with washing buffer (2 × SSC, 0.1 % SDS) at room temperature. Plates were then washed under more stringent conditions at 37 °C for 10 min. After four washes with washing buffer, 200 μl TB-TBS buffer (10 mM Tris-HCl, 0.5 M NaCl, pH 7.4, 0.5 % Tween 20, and 0.5 % blocking reagent; Roche Diagnostics, Basel, Switzerland) was added to block nonspecific binding. Next, 100 μl 1:2000-diluted alkaline-phosphatase-conjugated streptavidin in TB-TBS was added, and samples were incubated at 37 °C for 30 min. Plates were washed six times in T-TBS (10 mM Tris-HCl, 0.5 M NaCl, pH 7.4, 0.5 % Tween 20). For color development, 100 μl of substrate solution (4 mg/ml *p*-nitrophenylphosphate; Sigma Aldrich, St. Louis, Mo., USA, in 10 % diethanolamine, pH 9.8) was added, and absorbance was determined at 405 nm. The ratio of the hybridization intensity of each TCR V region (TCRV)-specific probe to that of a TCR-C-region-specific probe (V/C value) was determined using the TCR cDNA concentrated samples that contained the corresponding TCRV segment, and the universal TCR constant segment, respectively. Absorbance obtained for each TCRV-specific probe was divided by the corresponding V/C value. The relative frequency was calculated based on the corrected absorbance using the following formula: relative frequency (%) = (corrected absorbance of TCRV-specific probe/sum of corrected absorbance of TCRV-specific probes) × 100.

### Determination of CDR3 nucleotide sequences

PCR was performed with 1 μl of 1:20-diluted second PCR product, using a forward primer specific for the variable region and a reverse primer specific for the constant region (MCA4 or MCB4), under the conditions described above. Primers VA8-1 (5′-ACGCCACTCTCCATAAGAGCA-3′), VA10-1 (5′-GCTCTTTGCACATTTCCTCC-3′), and VB2-1 (5′-ACACGGGTCACTGATACGGA-3′) were used in this study. After elution from the agarose gel, PCR products were cloned into pGEM-T Easy Vector (Promega, Madison, WI, USA). DH5α competent cells were transformed with recombinant plasmid DNA. Sequence reactions were performed with a GenomeLab DTCS Quick Start Kit (Beckman Coulter) and analyzed using a CEQ8000 Genetic Analysis System (Beckman Coulter).

### Statistical analysis

Student’s *t*-test was used to assess statistical significance of changes in weight ratios. A log-rank test was performed to assess the survival curves of JEV-infected mice. One-way analysis of variance (ANOVA) followed by a Tukey test was used to assess statistical significance in TCR repertoire analysis and to evaluate CDR3 sequence frequency. A *p*-value <0.05 was considered statistically significant.

## Results

### JEV RNA levels in the brain and Th1 and Th2 cytokine balance in surviving and dying mice

**Fig. 1:**
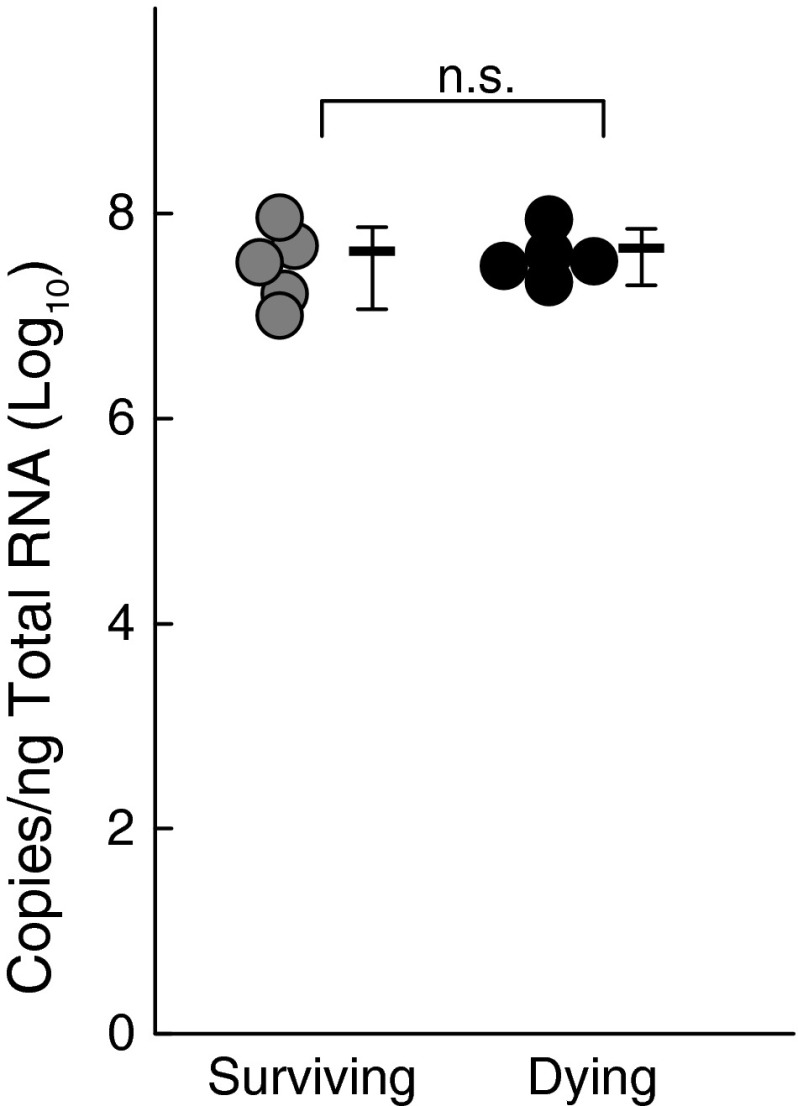
Comparison of JEV RNA levels in JEV-inoculated mice. Brain JEV RNA levels measured on day 13 after JEV inoculation. The circle indicates each individual measurement of RNA copies. A bar indicates the average of each group, and the vertical line indicates the range of SD

Two groups of five mice with brain virus titers of >1 × 10^6^ pfu/g were selected randomly from the dying and surviving groups as described in the Materials and methods section, and JEV RNA levels were measured (Fig. 1). There were no differences in the levels of brain JEV titers as assessed by RNA (Fig. 1) and pfu (data not shown) between the dying and surviving groups.

**Fig. 2:**
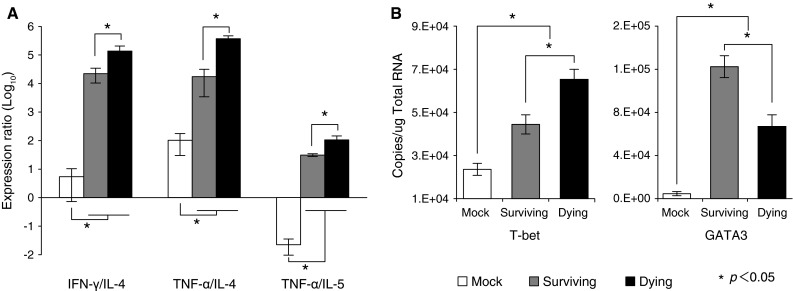
Expression ratio of Th1- to Th2-related transcription factors. (**A)** Ratio of IFN-γ/IL-4 (left), TNF-α/IL-4 (middle), and TNF-α/IL-5 (right) in JEV-infected mouse brain (**B**) mRNA expression level of Th1- and Th2-related transcription factors T-bet and GATA3. Significant differences (*p* < 0.05, ANOVA) between surviving and dying mouse brain at 13 days postinfection (dpi) are indicated by an asterisk (*)

We first analyzed the Th1 and Th2 balance in the brain after JEV inoculation by comparing expression levels of Th1-related and Th2-related genes (IFN-γ/IL-4, TNF-α/IL-4, and TNF-α/IL-5 ratios) in the brain on day 13 (Fig. 2A). The ratio of Th1- to Th2-related gene expression was higher in JEV-inoculated mice than in mock-infected mice. Among inoculated mice, these ratios were significantly higher in the dying group than in the surviving group. To determine the basis for this skewing, we measured the levels of two transcription factors, T-bet and GATA-3 (Fig. 2B). T-bet is an important activator of IFN-γ [29–31], while GATA-3 initiates IL-5 and IL-13 transcription [32–34]. T-bet and GATA-3 expression levels were higher in JEV-inoculated mice than in mock-infected mice on day 13. In addition, T-bet expression was significantly higher in dying mice than in surviving mice. In contrast, GATA-3 expression was significantly higher in the surviving group than in the dying group.

### Analysis of infiltrating cells and apoptosis-related markers

**Fig. 3:**
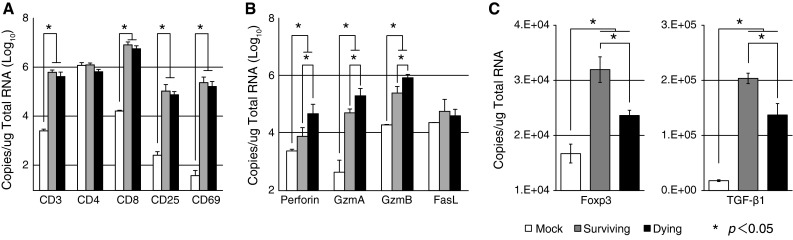
qRT-PCR quantification of mRNA expression of T-cell-related genes in the brain. RNA was extracted from brains of mock- and JEV-infected surviving and dying mice at 13 dpi (*n* = 5). (**A**) mRNA expression of CD3, CD4, CD8, CD25, and CD69 as T-cell-related antigens, (**B**) perforin, Gzm A, and Gzm B as cytotoxic granules, and (**C**) Treg-cell-related genes Foxp3 and TGFβ1 is shown. The mRNA expression levels in JEV-infected brains were normalized to GAPDH expression. Vertical error bars indicate the standard deviation (SD) of three independent experiments. Significant differences (*p* < 0.05, ANOVA) between surviving and dying mouse brain at 13 dpi are indicated by an asterisk (*)

To investigate the activation state of infiltrating T cells, we examined expression levels of T cell markers (CD3, CD4, CD8 and CD25), lymphocyte activation marker (CD69) (Fig. 3A), and apoptosis-related markers (perforin, Gzm A, Gzm B and FasL) (Fig. 3B). CD3, CD4, CD8 and CD25 are expressed on activated T cells and B cells [35], and CD69 is expressed rapidly after lymphocyte activation [36]. The expression levels of CD3, CD8, CD25, and CD69 were higher in JEV-infected mice than in mock-infected mice. However, expression levels of these cell-surface markers did not differ between the dying and surviving groups. In addition, CD4 transcripts were detected at similar levels in mock- and JEV-infected mouse brains, probably because of overexpression on microglia cells. The expression levels of apoptosis-related molecules such as perforin, and Gzm A and B were higher in the dying group than in the surviving group.

We next examined expression levels of the Treg-related genes TGF-β1 and Foxp3 in JEV-infected mice (Fig. 3C). TGF-β1 is a major pluripotent cytokine with a pronounced immunosuppressive effect [37]. Foxp3 is specifically expressed in Treg cells, and its expression is essential for the programming of Treg cell development and function [38–41]. Interestingly, TGF-β1 and Foxp3 levels were higher in JEV-infected mice, and among infected mice, levels were higher in mice that survived.

### TCR usage in brain-infiltrating T cells

**Fig. 4:**
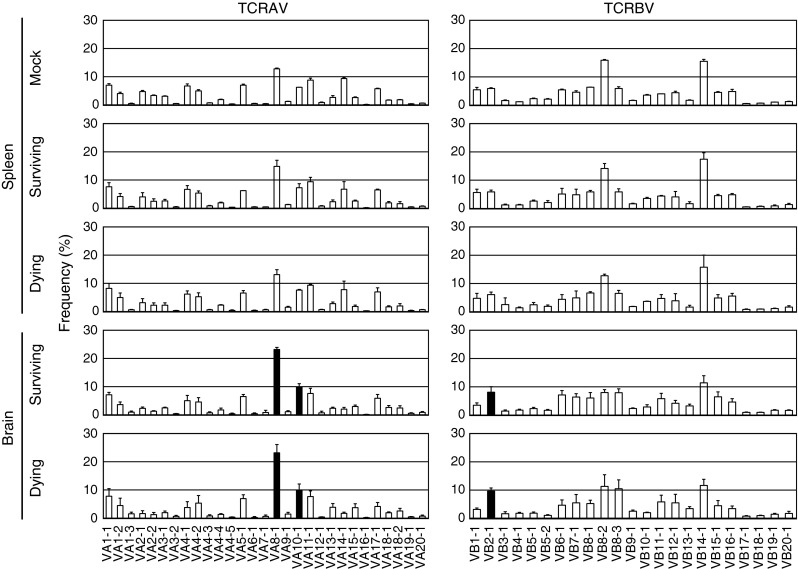
TCR repertoire analysis of spleen and brain from mock- or JEV-infected mice. TCR VA and TCR VB repertoires were analyzed by MHA as described in Materials and methods. Mean percent frequencies ±SD of five mice are indicated. Black bars indicate a significant increase compared with mock-infected spleen (*p* < 0.05, ANOVA)

We have previously demonstrated that the number and activation state of brain-infiltrating CD3+CD8+ T cells were similar between the dying and surviving groups, but that cytokine profiles were different. We analyzed TCR VA and TCR VB repertoires of infiltrating T cells (Fig. 4), where spleens from mock-infected mice served as controls. The number of T cells in the brains of mock-infected mice was low, and it was therefore not possible to determine TCR usage of these T cells. The spleen was used as a control, because there were no significant difference in TCR usage in spleens between the surviving and dying groups. The frequency of T cells bearing VA8-1, VA10-1, and VB2-1 was higher in JEV-infected brains than in mock-infected spleen. VA14-1, expressed on NKT cells [42], was expressed at equally low levels in the dying and surviving groups, and we did not detect TCR VA or TCR VB expression in mock-infected mice due to low lymphocyte numbers (data not shown).

### Differences in CDR3 amino acid sequences of highly expressed TCRs from brain-infiltrating T cells between surviving and dying groups

**Fig. 5:**
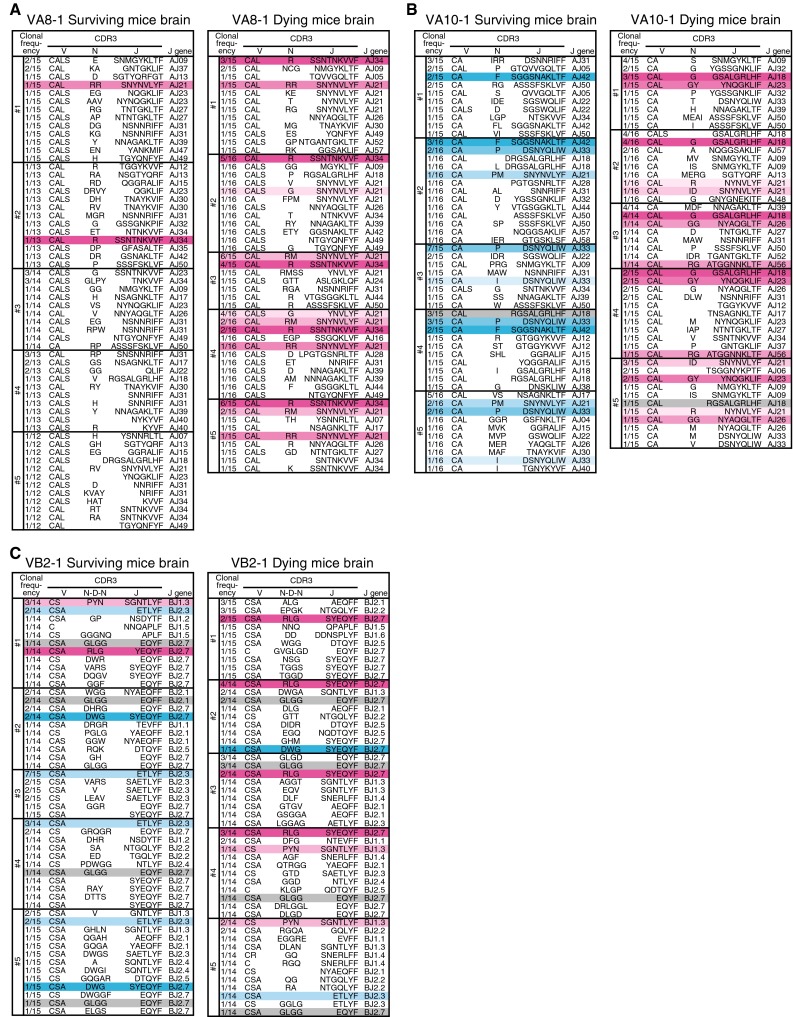
Amino acid sequences of TCR CDR3 regions of cDNA clones derived from JEV-infected brain. Predicted amino acid sequences are shown with their frequencies of cDNA clones from each mouse for the (**A**) VA8-1, (**B**) VA10-1, and (**C**) VB2-1 families. J gene usage is shown at the right side of each sequence. V, N (N-D-N), and J gene segments are not strictly divided. Each color indicates a group of identical sequences

The nucleotide sequences of the VA8-1, VA10-1, and VB2-1 CDR3 regions were determined using PCR and randomly selected cDNA clones. Predicted amino acid sequences are shown along with the frequency of cDNA clones isolated from the brains of JEV-infected mice (Fig. 5). We analyzed more than 30 clones in mock-infected spleens but did not find any clones with an identical sequence (data not shown).

The same VA8-1 CDR3 sequences (CAL-R-SSNTNKVVF, CAL-RM-SNYNVLYF, CALS-G-SNYNVLYF and CAL-RR-SNYNVLYF) were detected in the brains from all five mice that died (Fig. 5A). Two sequences, CAL-R-SSNTNKVVF and CAL-RR-SNYNVLYF, were each detected once in the surviving mice.

The following VA10-1 CDR3 sequences (CAL-G-GSALGRLHF, CAL-R-NYNVLYF, CAL-GY-YNQGKLIF, CAL-GG-NYAQGLTF, CAL-RG-ATGGNNKLTF and CA-ID-SNYNVLYF) were detected in two or more dying mice (Fig. 5B). The sequences (CA-F-SGGSNAKLTF, CA-P-DSNYQLIW, CA-PM-SNYNVLYF, and CA-I-DSNYQLIW) were detected in two or more surviving mice. Only one CDR3 sequence (CAL-RGSALGRLHF) was detected in both surviving and dying mice.

Two VB2-1 CDR3 sequences (CSA-RLG-SYEQYF and CS-PYN-SGNTLYF) were detected mainly in dying mice, while two others (CSA-ETLYF and CSA-DWG-SYEQYF) were detected mainly in surviving mice (Fig. 5C). One CDR3 sequence (CSA-GLGG-EQYF) was detected commonly in both groups.

**Fig. 6:**
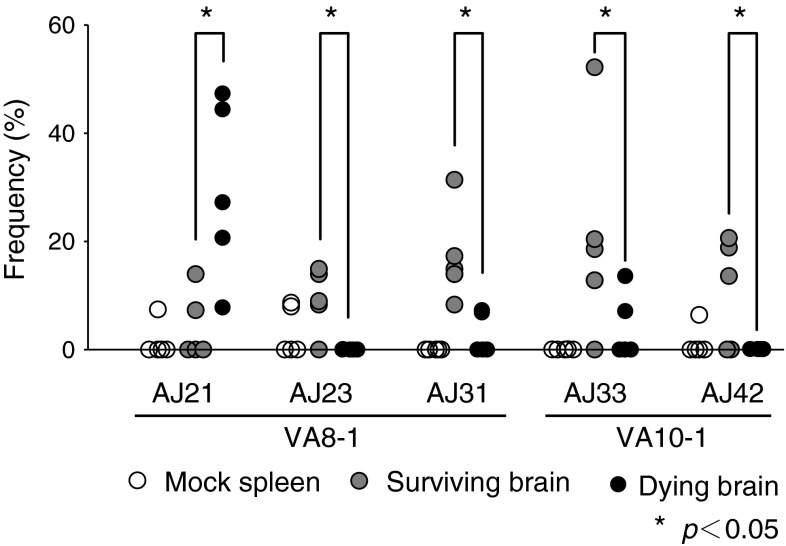
Differential patterns of TCR J gene usage between surviving and dying mice. Frequencies of characteristic V/J combinations are plotted individually. Open circles indicate spleen of mock-infected mice. Filled gray circles indicate brain of surviving mice. Filled black circles indicate brain of dying mice. An asterisk indicates a significant (*p* < 0.05, ANOVA) difference between the surviving and dying groups

Several reports have shown that the CDR3 β-chain J region does not interact specifically with antigenic peptides [43–47].We also examined J gene usage in T cells with VA8-1 or VA10-1 (Fig. 6). The frequencies of VA8-1/AJ23, VA8-1/AJ31, VA10-1/AJ33, and VA10-1/AJ42 were significantly higher in the surviving group than in the dying group. In contrast, VA8-1/AJ21 was more prevalent in the dying group than in the surviving group. These results indicate that although there were no quantitative differences in the number or activation state of infiltrating CD8+ T cells between the dying and surviving groups, there were qualitative differences in TCR usage. The results suggest that the qualitative differences affect the outcome of JEV infection: death or survival.

## Discussion

It is generally understood that the levels of JEV in the brain determines the outcome of JEV infection in mice; high JEV levels in the brain lead to fatal outcome, while low JEV levels lead to survival. Although high levels of JEV (>1 × 10^6 ^pfu/g) were detected on day 13 in all dying mice, the levels of JEV were variable in surviving mice; some had JEV levels higher than 1 × 10^6^ pfu/g, but others had low levels. Thus, the results were in part consistent with the general understanding of the pathogenesis of JEV infection that the level of JEV in the brain determines the outcome. However, the results also indicate that the outcome was not determined solely by the JEV level in the brain. In order to define the mechanisms that lead to fatal outcome or survival, we attempted to compare two groups of mice with similarly high brain JEV levels: a group of five dying mice with >1 × 10^6^ pfu/g and a group of five surviving mice with >1 × 10^6^ pfu/g. In the present study, we used C57BL/6j (B6) mice as the model, and the results suggest that the outcome of JEV infection is not determined solely by JEV levels in the brain in C57BL/6j (B6) mice.

Our results are also consistent with those obtained using mice infected with tick-borne encephalitis virus (TBEV), where the intracerebral viral load was similar in mice that died and in those that survived [24]. TBEV elicits dose-dependent mortality following peripheral infection in some mouse strains [48, 49]. Furthermore, weight loss is an indicator of a fatal outcome, and the degree of weight loss may be a simple and effective marker for evaluating JEV-infected mice, allowing the discrimination of mice that will survive from those that will die [49]. These results suggest that the outcome of JEV infection in C57BL/6j (B6) mice is not determined by the JEV level in the brain. It has been reported that CD8^+^ T cells play an important role in viral clearance in the brain [50–53]. Although a CD8^+^ T cell response is required to clear flavivirus infection [50, 53], an excessive response may cause immunopathology [5]. In the present study, there were no significant differences in the number of infiltrating T cells or their activation state in the brain as determined by CD antigen expression. This suggests that qualitative differences of intracerebral infiltrating T cells may be a factor in determining outcomes. We have previously reported that CD8^+^ T cells infiltrated mainly the brain of TBEV-infected mice, and quantitative differences in the number of T cells infiltrating the brain did not appear to be a factor in mouse survival [24, 49].

TCR repertoire analysis of infiltrating T cells revealed that the frequencies of T cells bearing VA8-1, VA10-1, and VB2-1 increased significantly in brains of JEV-infected mice. However, their frequency increased equally in mice that survived and those that died. These results indicate that once a certain amount of virus is inoculated, T cells expressing selected TCR V families accumulate in the brain. Interestingly, CDR3 sequence analysis provides a means of differentiating between surviving and dying mice. Analysis of CDR3 amino acid sequences of infiltrating T cells revealed that dominant clones bear the same VA or VB type (VA8-1, VA10-1 and VB2-1). Subsequently, AJ21 was elevated in the brains of mice that died, and AJ23 and AJ31 were elevated in mice that survived, and the observed J use frequency was confirmed. Infiltrating T cell clones from the brain were different between surviving and dying mice, even though they were subjected to the same JEV infection. In our previous report on CDR3 sequences present following TBEV-infection, a clear clonal difference in the brain between dying and surviving mice was not observed. There are probably differences in immune induction such as the early immune response that balance antibody-mediated or cell-mediated immunity in JEV and TBEV infection. CD4 transcripts were persistently overexpressed in both mock- and JEV-infected mouse brain. This is probably due to the presence of microglia that express CD4 in CNS-resident cells [54, 55].

Tregs suppress effector T cells to prevent or control reactivity to self-antigens [56] and pathogens [57], to blunt inflammation [58], and to maintain antigen-specific T-cell homeostasis [59]. Tregs have been defined as CD3^+^CD4^+^CD25^+^, shown to express Foxp3, and play a role in immune tolerance [37]. The peripheral Treg frequency influences post-infection symptom development after West Nile virus infection [53]. Because peripheral blood analysis may not fully reflect the sites of infection, and lower peripheral Treg levels may not correlate with tissue levels, it is important to examine intracerebral Treg levels [16, 60, 61]. We measured mRNA expression of inflammatory cytokines, cell-surface antigens, and the Treg-related factors TGF-β1 and Foxp3 using qRT-PCR. Treg RNA levels were higher in the brain of surviving mice than in dying mice. In infections caused by hepatitis C virus, another member of the family *Flaviviridae*, there is an inverse correlation between Treg number in the periphery of liver biopsies and the histological inflammatory score [62]. Our results suggest that Treg inhibits intracerebral cell-mediated immunity. The levels of the cytotoxic factors perforin and Gym A and B were elevated in the brains of dying mice relative to surviving mice, consistent with the elevation of Treg-related genes. The intracerebral cytokine balance was biased toward a Th1 profile in dying mouse brain compared with surviving mouse brain. This is also consistent with results related to the epistatic transcription factors GATA3 and T-bet. Since a correlation between the expression of Treg genes and the level of JEV-specific T cells was observed in mouse brain, we speculate that Tregs may exert a protective effect by suppressing the JEV-specific immune response and inflammation. It will therefore be important to determine whether the T cells accumulating in the brain provide positive or negative effects on infection outcome. Future studies will be needed to determine the contribution of innate immunity and the mechanisms of induction and action of Tregs.

In conclusion, we have described a qualitative association between brain-infiltrating T cell clones and disease severity in JEV-infected mice. In addition, certain clones were inhibited in the surviving mouse group by Tregs, and there was a bias towards a Th2 cytokine profile in the brain compared with mice that died. Thus, the downregulation of an effective response by Tregs may be critical for mitigating bystander injury and disease pathology in the CNS. It will be interesting to determine what leads to Treg activation, which may provide new hints at pharmacologic control of viral pathogenesis.
